# Genetic variants in *NECTIN4* encoding an adhesion molecule are associated with continued opioid use

**DOI:** 10.1371/journal.pone.0234549

**Published:** 2020-06-18

**Authors:** Chiu-Ping Fang, Tung-Hsia Liu, Ren-Hua Chung, Hsiao-Hui Tsou, Hsiang-Wei Kuo, Sheng-Chang Wang, Chia-Chen Liu, Shu Chih Liu, Andrew C. H. Chen, Yu-Li Liu

**Affiliations:** 1 Center for Neuropsychiatric Research, National Health Research Institutes, Zhunan, Miaoli County, Taiwan; 2 Division of Biostatistics and Bioinformatics, Institute of Population Health Sciences, National Health Research Institutes, Miaoli County, Taiwan; 3 Graduate Institute of Biostatistics, China Medical University, Taichung, Taiwan; 4 Department of Psychiatry, the Zucker Hillside Hospital, Northwell Health, Glen Oaks, New York, United States of America; 5 The Feinstein Institute for Medical Research, Hofstra Northwell School of Medicine at Hofstra University, Manhasset, New York, United States of America; 6 Graduate Institute of Clinical Medical Science, China Medical University, Taichung, Taiwan; Tokyo Metropolitan Institute of Medical Science, JAPAN

## Abstract

Methadone is a synthetic opioid used as maintenance treatment for patients addicted to heroin. Skin irritation is one of the adverse events caused by opioid use. 344 methadone maintenance treatment (MMT) patients were recruited with records and measurements on methadone dose, plasma methadone concentrations, and treatment emergent symptom scales (TESS). 15 patients reported with skin irritation. Five SNPs located within the *NECTIN4* genetic region were genotyped. The *NECTIN4* gene within the adherens junction interaction pathway was associated with methadone dose in pathway-based genome wide association analyses (*P* = 0.0008). Three highly-linked SNPs, rs11265549, rs3820097, and rs4656978, were significantly associated with methadone dose (*P* = 0.0003), plasma concentrations of *R*,*S*-methadone (*P* = 0.0004) and TNF-α (*P* = 0.010) in all 344 MMT patients, and with self-report skin irritation symptom scores (*P* = 0.010) in the 15 MMT patients who reported with skin irritation. To identify the possible roles of plasma level of Nectin-4 in the responses to MMT and opioid use, additional age- and gender-matched 51 controls and 83 methadone-free abstinent former heroin users were recruited. Plasma level of Nectin-4 was the highest in MMT patients among the three groups. The results suggest involvement of genetic variants on *NECTIN4* in methadone dose. Plasma Nectin-4 level is likely an indicator for continued use of opioids.

## Introduction

Methadone is a synthetic opioid commonly prescribed for heroin dependent patients as a maintenance therapy [1]. The mechanism of action has been well documented through its binding to the mu-opioid receptor as a full agonist [2]. Multiple candidate genes have been found to be associated with methadone dosage, for example, metabolic enzyme genes *CYP2C19* [3, 4], *CYP2D6* [5], *CYP3A4* [4], *CYP2B6* [4, 6], ATP-binding cassette (ABC) transporter gene *ABCB1* [5, 6], opioid receptor gene *OPRM1* [7], and dopaminergic receptor subtype gene *DRD2* [8]. A few pathways have been reported to be associated with methadone dosage, for example, neurotrophin of *BDNF* and *NTRK2* [9], opioid receptor pathway of *OPRM1* [9], and dopaminergic pathway of *DRD2* and *ANKK1* [9]. In our previous report using gene-based genome-wide association analyses on methadone maintenance treatment (MMT) in heroin dependent patients, G protein-coupled receptor kinase 5 (*GRK5*), which is one of the genes regulating the desensitization of the mu-opioid receptor, has demonstrated a strong association with methadone dosage [10]. An association has also been observed between increase in methadone dosage and elevation of plasma TNF-α level [11].

Skin irritation is one of the adverse events caused by opioid use. This response caused by histamine release is usually treated as an allergic inflammatory response to opioids [12, 13]. The irritation response has also been recognized as a response related to opioid receptors located both in the central and peripheral nervous systems [14]. Skin irritation response is usually not life threatening, and often disappears following discontinuation of opioid use.

Nectin-4, encoded by *NECTIN4* (*nectin cell adhesion molecule 4*) gene, is a member of the nectin family that is involved with cell adhesion [15]. It consists of a single-pass type I membrane protein, an epithelial cell receptor for Morbilliviruses, and a secreted form, produced by proteolytic cleavage through the metalloproteinase ADAM17/TACE [16]. *NECTIN4* is highly expressed in the skin (https://www.ncbi.nlm.nih.gov/gene/81607/?report=expression). Mutations in *NECTIN4* may cause ectodermal dysplasia-syndactyly syndrome (EDSS1), which is an autosomal disorder [17–19]. In our previous study, we found that plasma cadherin 2 (CDH2) was associated with methadone dose change [20]. Both *CDH2* and *NECTIN4* genes belong to the adherens junction interaction pathway in the pathway-based association analyses. In this study, we evaluated the role of human nectin family member *NECTIN4* gene for its role in methadone dose. We found that the genetic variants in the *NECTIN4* gene were associated with methadone dosage, and plasma *R*,*S*-methadone concentrations. Moreover, the plasma Nectin-4 level was higher in subjects who continued using opioid than those who either never abused opioid or had quit heroin use. The results suggested that the *NECTIN4* genetic variants are likely an indicator for methadone dose, and Nectin-4 is related to the continued use of opioid.

## Materials and methods

### Methadone maintenance subjects

This study was approved by the institutional review boards of the National Health Research Institutes (EC0970504, Zhunan, Taiwan) and the seven participating hospitals [21]. All participants had singed the informed consent. The registered number of the project for 344 MMT patients in the National Institutes of Health (NIH) Clinical Trial was NCT01059747. The 344 MMT patients passed the genome-wide association study (GWAS) quality controls with available detailed data regarding plasma methadone concentrations [22]. The inclusion and exclusion criteria were described in our previous report [21].

The study protocol for normal controls and former heroin user subjects was approved by the Institutional Review Board of the National Health Research Institutes (EC0980209-R5, Zhunan, Taiwan). All participants had singed the informed consent. The inclusion and exclusion criteria for these two subjects were described in our previous report [10]. The registered number of the project in the National Institutes of Health (NIH) Clinical Trial was NCT01668706.

### Clinical assessments

Demographics, clinical characteristics and methadone treatment course, including the dose and treatment duration and the treatment adherence over the previous week, were obtained from the medical records of participating subjects. Information regarding administration with other medications in the previous week was obtained either from medical records and self-report by subject. Methadone-related adverse events were assesses by research nurses using the Treatment Emergent Symptoms Scale (TESS) [23]. The TESS consists of 43 treatment emergent symptoms. The higher was the symptom score, the severer were the clinical symptoms. It was counted as adverse events related to methadone only if the symptoms occurred after the initiation of MMT. The severity of each symptom was rated on a 3-point Likert scale ranging from mild, moderate, to severe.

### *NECTIN4* SNP selection and genotyping

The Axiom Genome-Wide CHB 1 Array, which is population-optimized with a better coverage of common genomic alleles (minor allele frequency, MAF > 5%) for Han Chinese, was employed for genome-wide genotyping. Two single nucleotide polymorphisms (SNPs), rs11265549 and rs3892375, were located within *NECTIN4* genetic loci in the genome-wide genotyping database [22]. The raw data can be accessed in Gene Expression Omnibus (GEO accession number: GSE78098) [22]. For a better coverage of SNPs in the *NECTIN4* gene, additional SNPs, rs4656978 and rs3820097, were further selected and genotyped by matrix-assisted laser desorption/ionization- time of flight mass spectrometry at the National Center for Genome Medicine (NCGM), Taiwan. SNP rs12116949 at 3’UTR was genotyped with TaqMan SNP Genotyping Assays (Applied Biosystems, USA).

### Analyses of plasma methadone concentration

12 ml whole blood was collected from subjects before the next methadone dose was given, when the plasma concentration of methadone is likely to be at its lowest level. After centrifugation at 2000 × g in a Kubota 2800 centrifuge (Kubota Co., Osaka, Japan) for 20 min at 4 °C, the plasma was collected from the supernatant of whole blood and dispensed into a 1 mL/microcentrifuge tube then frozen at −80 °C until use. Plasma concentrations of racemic methadone were measured using high-performance liquid chromatography (HPLC) with the method described in our previous report [24].

### Plasma Nectin-4 assay

A quantitative sandwich enzyme immunoassay technique (R&D Systems, Minneapolis, MN) was used to detect soluble Nectin-4 in plasma of MMT patients. A monoclonal antibody specific for human Nectin-4 has been pre-coated onto a microplate. 50 μl of standards and samples were pipetted into the wells and incubated for 2 hours at 2–8°C. After washing away any unbound substances, a cold enzyme-linked polyclonal antibody specific for human Nectin-4 was added to the wells for 2 hours at 4°C. Following a wash to remove any unbound antibody-enzyme reagent, a substrate solution was added to the wells for 30 minutes at room temperature and the dense of color developed in proportion to the amount of Nectin-4 bound in the initial step. After the stop solution was added, plates were read for absorption at 450 nm and 570 nm, and concentrations of Nectin-4 in plasma were calculated from standard curves. The detection limit was 16.6 pg/ml for Nectin-4. 33 out of 344 MMT patients did not have data for plasma Nectin-4 level due to lack of plasma samples.

### Plasma TNF-α assay

Plasma concentration of cytokine tumor necrosis factor-α (TNF-α) was measured using the Milliplex MAP human cytokine/chemokine magnetic bead panel kit (Millipore, Billerica, MA) according the manufacturer protocol. Data were acquired by a MAGPIX Multiplex Reader (Luminex Corp., Austin, TX). 5 out of the 344 MMT patients did not have data for plasma levels of TNF-α due to lack of plasma samples.

### Statistical analyses

Methadone treatment responses between the MMT patients with or without skin irritation were compared by the Mann-Whitney U test for continuous variables and the Fisher’s exact test for categorical variables using the SAS software, Version 9.4 (SAS Institute, Inc., Cary, NC). The pathway-based and gene-based association analyses were performed based on the Knowledge-based mining system for Genome-wide Genetic studies (KGG, Version 2.5). The pathway-based *P*-values were calculated using the Hybrid set-based test (HYST) [25] and the gene-based *P*-values were calculated using the extended Simes test (GATES) [26] in KGG. The associations of single nucleotide polymorphisms (SNPs) in the *NECTIN4* gene with methadone dose, plasma methadone concentrations, plasma TNF-α level, and skin irritation were tested by the correlation/trend tests. Multiple comparisons were considered with the false discovery rate (FDR) using SNP & Variation Suite, Version 8.4.0 (Golden Helix, Inc., Bozeman, MT). The body weight was considered as a confounding factor of methadone dose by the General linear model (GLM) using the SAS software, Version 9.4 (SAS Institute, Inc., Cary, NC). The comparisons among age- and gender-matched controls, medication-free abstinent former heroin users (abstinent) and MMT patients were calculated by the nonparametric Kruskal Wallis test, Mann-Whitney U test and the contingency table of Chi-square test and Fisher’s exact test. Multiple comparisons among the plasma Nectin-4 levels were performed by Mann-Whitney U test, and correlation between plasma Nectin-4 with age were analyzed by the spearman correlation analyses, were plotted and analyzed by GraphPad Prism 5 (GraphPad Software, San Diego, CA, USA). To explore the predicting cut-off value for the plasma level of Nectin-4 in the age- and gender-matched controls and MMT patients with or without skin irritation, receiver operating characteristic (ROC) analyses were conducted and the area under the curve (AUC) with an associated 95% confidence interval (CI) was calculated. The ROC curves were plotted by GraphPad Prism 5 (GraphPad Software, San Diego, CA, USA). HAPLOVIEW version 4.2 [27] was used for Hardy-Weinberg equilibrium tests and Tagger algorithm. A *P*-value less than 0.05 was set as threshold for statistical significance.

## Results

### Characteristics of the methadone maintenance treatment patients

The enrolled 344 MMT patients had an average age of 38 years and 81.69% were male. The average methadone dosage was 55.22 mg/day. Concentration of *R*,*S*-methadone in plasma was 336.96 ng/ml. Plasma level of cytokine TNF-α was 10.55 pg/ml. Plasma Nectin-4 concentration was 238.31 pg/ml. Fifteen patients (around 4.4% in 344 MMT patients) self-reported with skin irritation after taking methadone (Table 1).

**Table 1:** General demography of the methadone maintenance treatment patients in this study.

Variable	n	Mean ± SD
Age (year)	344	38.16 ± 7.69
BMI	341	23.64 ± 3.52
Gender		
Male	281	(81.69%)
Female	63	(18.31%)
Skin Irritation of side effect	15	1.47 ± 0.83
Methadone dosage (mg/day)	344	55.22 ± 28.47
*R*-Methadone (ng/ml)	344	194.44 ± 123.56
*S*-Methadone (ng/ml)	344	142.52 ± 99.64
*R*,*S*-methadone (ng/ml)	344	336.96 ± 212.72
TNF-α (pg/ml)	339	10.55 ± 8.68
Nectin-4 (pg/ml)	311	238.31 ± 79.64

SD, Standard deviation.

### Adherens junction interactions pathway is associated with methadone dose

The adherens junction interactions pathway was found to be the pathway most significantly associated with methadone dose by screening a total of 1,421 pathways using Knowledge-based mining system for Genome-wide Genetic studies (KGG, Version 2.5, *P* = 0.0001) (S1 Table). Although this pathway did not pass the threshold for statistical significance in the genome-wide pathway-based association analyses (*P* = 0.00004), the *NECTIN4* gene in the pathway showed the most significant association with methadone dose (*P* = 0.0008). We therefore compared the associations between the genetic variants in the *NECTIN4* with methadone dose and its treatment responses.

### *NECTIN4* gene associated with methadone dose and its plasma concentration

Five SNPs in the genetic loci of *NECTIN4* were genotyped (S2 Table). All five SNPs had passed the Hardy-Weinberg’s equilibrium test with P-value above 0.05. 3 out of the 5 SNPs, rs11265549 (intron 1), rs3820097 (intron 2), and rs4656978 (intron 6), showed a highly linkage disequilibrium (D’ = 1, and linkage disequilibrium correlation r^2^ > 0.97) (S1 Fig). rs11265549 was selected as the tagger representing rs3820097 and rs4656978 by the Tagger algorithm in HAPLOVIEW. The three highly-linked SNPs, rs11265549, rs3820097, and rs4656978 showed significant associations with the methadone dose and plasma methadone concentrations, but not rs3892375. The number of subjects who carried the minor genotype of rs3892375 or rs12116949 was no greater than 2 (Table 2). The minor genotype carriers of rs11265549 had a lower methadone dose (*P* = 0.0003) and lower plasma *R*,*S*-methadone concentration (*P* = 0.0004) than the major genotype carriers (Table 2). The minor allele type carriers also had a lower methadone dose (*P* = 0.0002) and lower plasma *R*,*S*-methadone concentration (*P* = 0.0003) than the major allele type carriers (S3 Table). The associations between *NECTIN4* genetic variants with *R*-form and *S*-form methadone are in a similar pattern as shown in the S4 Table.

**Table 2:** *NECTIN4* is associated with methadone dosage (mg/day) and plasma concentrations (ng/mL) of *R*,*S*-methadone.

SNP_ID	Genotype	Methadone dosage (mg/day)				Plasma *R*,*S*-methadone concentration (ng/mL)			
		N	Mean ± SD	*P*-value (Adjusted)	FDR	N	Mean ± SD	*P*-value	FDR
rs3892375 (Intron 1)	AA	283	54.19 ± 28.56	0.149	0.149	283	331.05 ± 208.22	0.267	0.267
	AG	60	59.25 ± 27.41	(0.065)		60	362.91 ± 233.99		
	GG	1	105.00 ±.			1	451.60 ± .		
rs11265549 (Intron 1)	GG	144	61.40 ± 29.79	**0.0003**	**0.0007**	144	381.43 ± 239.76	**0.0004**	**0.0006**
	AG	153	52.39 ± 27.63	(**0.002**)		153	316.73 ± 186.14		
	AA	46	45.76 ± 22.99			46	266.64 ± 179.69		
rs12116949 (3' UTR)	CC	255	53.11 ± 28.55	**0.020**	**0.024**	255	319.06 ± 200.26	**0.017**	**0.021**
	AC	87	61.21 ± 27.72	(0.067)		87	392.59 ± 239.72		
	AA	2	65.00 ± 21.21			2	199.93 ± 59.36		

SD, standard deviation. *P*-value, Trend/Correlation analysis of *p*-value.

Parenthesis adjust, *p*-value adjusted body weight with general linear model.

FDR, False Discovery Rate. Bold values indicate P < 0.05.

rs11265549 was selected as the tagger SNP representing rs3820097 and rs4656978 by the Tagger algorithm in HAPLOVIEW.

### *NECTIN4* gene is associated with plasma TNF-α level and skin irritation

The three highly-linked SNPs, rs11265549, rs3820097, and rs4656978 were associated with plasma TNF-α levels in all 344 MMT patients (*P* = 0.010) and skin irritation identified by the TESS symptom scores in the 15 MMT patients (*P* = 0.010) (Table 3). The minor genotype carriers of these three highly-linked SNPs, who had a lower methadone dose and plasma methadone concentration, had a higher plasma level of TNF-α and higher skin irritation TESS scores than the major genotype carriers. The minor allele type carriers also had higher plasma TNF-α levels and higher skin irritation TESS scores than the major allele type carriers (S5 Table).

**Table 3:** *NECTIN4* genetic variants are associated with plasma TNF-α level and skin irritation in TESS scores.

SNP_ID	Genotype	Plasma TNF-α (pg/ml)				Skin irritation			
		N	Mean ± SD	*P*-value	FDR	N	Mean ± SD	*P*-value	FDR
rs3892375 (Intron 1)	AA	278	10.76 ± 9.40	0.358	0.358	15	1.47 ± 0.83	-	-
	AG	60	9.58 ± 4.06			0	.± .		
	GG	1	10.66 ± .			0	.± .		
rs11265549 (Intron 1)	GG	144	9.62 ± 5.41	**0.010**	**0.024**	5	1.00 ± 0.00	**0.010**	**0.014**
	AG	149	10.41 ± 6.91			8	1.38 ± 0.74		
	AA	45	13.86 ± 17.57			2	3.00 ± 0.00		
rs12116949 (3' UTR)	CC	250	10.85 ± 9.66	0.312	0.358	13	1.54 ± 0.88	0.423	0.423
	AC	87	9.67 ± 5.00			1	1.00 ± .		
	AA	2	11.30 ± 0.59			1	1.00 ± .		

SD, standard deviation.

*P*-value, Trend/Correlation analysis of *p*-value.

FDR, False Discovery Rate.

Bold values indicate P < 0.05.

### MMT patients showed higher plasma Nectin-4 levels than controls and abstinent

The *NECTIN4* gene encodes an adhesion protein Nectin-4 involving mechanisms of cell-cell interactions. Part of the Nectin-4 protein may be released into the plasma after ADAM17/TACE enzyme catalysis. To further confirm the role of Nectin-4 in opioid addition, we examined the plasma Nectin-4 levels among the age (±3 years)- and gender-matched controls, medication-free abstinent former heroin users, and MMT patients (S6 Table). The abstinent former heroin users had a higher average BMI than MMT patients and controls. The MMT patients showed a more severe cigarette smoking status, where the plasma nicotine metabolite cotinine levels were significantly higher in the patients than the controls and former heroin users. Levels of aspartate aminotransferase (AST) and alanine aminotransferase (ALT), which represented liver function, were higher in MMT patients and former heroin users than the controls. This could reflect the status of hepatitis C virus (HCV) infection: the HCV infection rates were 100% and 92% respectively in MMT patients with and without skin irritation, 80% in former heroin users, and 0% in the controls.

**Fig 1:**
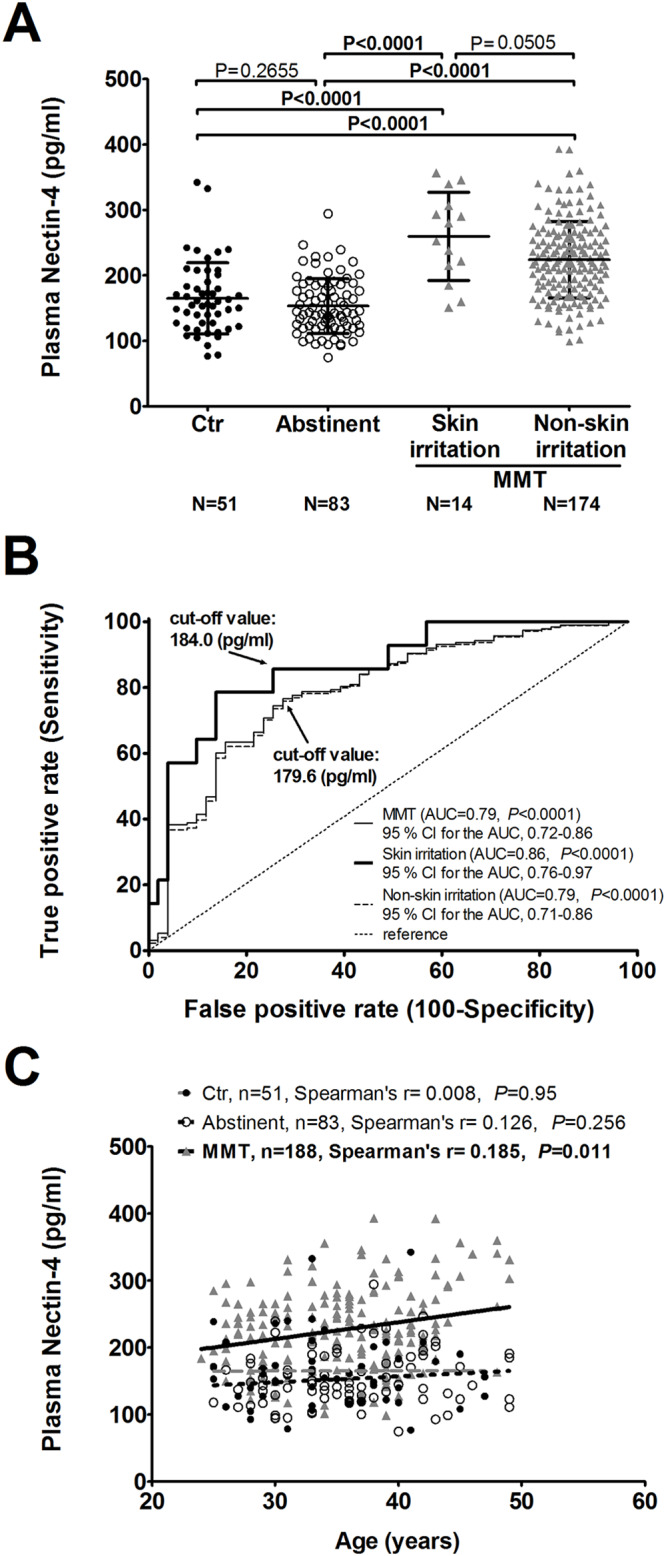
(A) The plasma Nectin-4 levels among age- and gender-matched normal controls (Ctr), medication-free abstinent former heroin users (Abstinent), MMT patients with self-report of skin irritation (Skin irritation) and without skin irritation (Non-skin Irritation). (B) A suggested cut-off level in correlation with plasma level of Nectin-4 was shown using receiver operating characteristic (ROC) curve analyses. AUC, Area under the curve. CI, Confidence interval. (C) The plasma Nectin-4 levels were correlated with age in age- and gender-matched MMT patients, but not in normal controls (Ctr) and medication-free abstinent former heroin users (Abstinent).

The rank of plasma levels of Nectin-4 is as follows: MMT patients with skin irritation > MMT patients without skin irritation (*P* = 0.051) > controls (*P*<0.0001) = former heroin users (*P*<0.0001) (Fig 1A). The plasma levels of Nectin-4 at the cut-off value 184 (pg/ml) in controls and MMT patients with skin irritation showed the highest level of sensitivity (85.7%) and specificity (74.5%) (AUC = 0.86, *P* < 0.0001) than MMT patients without skin irritation and MMT patients in the ROC curve analyses (Fig 1B). The plasma Nectin-4 levels showed a positive correlation with age (r = 0.265, *P*<0.0001) and the addiction duration (r = 0.259, *P*<0.0001) in all 344 MMT patients. The association with age was observed only in MMT patients (r = 0.185, *P* = 0.011) but not in the age- and gender- matched controls, or methadone-free abstinent former heroin users (Fig 1C). None of these five SNPs on *NECTIN4* gene showed association with the plasma level of Nectin-4.

## Discussion

Nectins are a group of adhesion molecules responsible for the cell-cell interaction through a calcium-independent adhesion mechanism [28]. In this study, we identified that the *NECTIN4* gene, one of such genes in the human expression nectin family, showed the most significant association with methadone dose among the adherens junction interactions in the pathway-based association analyses (*P* = 0.0008) (S1 Table), although it did not pass the pathway threshold. The results were further verified by three additional highly-linked SNPs located within the *NECTIN4* genetic loci, rs11265549 (intron 1), rs3820097 (intron 2), and rs4656978 (intron 6), which also showed significant associations with methadone dose and plasma *R*,*S*-methadone concentrations (Table 2). The minor genotype carriers of the three highly-linked *NECTIN4* SNPs had a lower methadone dose and lower plasma *R*,*S*-methadone concentrations than the major genotype carriers. These three highly-linked SNPs did not show interactions with *GRK5* gene, which is the gene showing the most significant level of association with methadone dose in our genomewide study [10]. This may explain in part why these three highly-linked SNPs on *NECTIN4* gene associated with methadone were not identified with the GRK5 pathway.

In terms of tissue gene expression distribution, *NECTIN4* is highly expressed in the skin [17]. The observation is aligned with our findings in the present study that the same three highly-linked SNPs rs11265549, rs3820097, and rs4656978 in the *NECTIN4* gene were also associated with skin irritation in MMT patients (Table 3), despite that only 15 out of 344 MMT patients in our cohort self-reported skin-irritation adverse reaction. The small number of subjects may affect the statistical power. It was shown that the three highly-linked SNPs demonstrating significant associations in 344 MMT patients did not show association with methadone dose, nor with plasma TNF-α level if analyzed only within the 15 patients reported with skin irritation due to a small sample size. The genotype and allele types of these three highly-linked SNPs were associated with methadone dose, methadone plasma concentration, and level of TNF-α when all 344 MMT patients were included for analyses.

The extracellular domain protein encoded by the *NECTIN4* gene can be catalyzed by the metalloproteinase ADAM17/TACE enzyme [16]. The released extracellular *NECTIN4* levels were measured through the plasma of all MMT patients in this study. These three highly-linked *NECTIN4* SNPs mentioned above did not show significant associations with plasma levels of Nectin-4. This suggested that the plasma Nectin-4 level may be mainly regulated by the ADAM17/TACE enzymatic activity. The ADAM17/TACE activity can be regulated by several factors. For example, the sheddase activity of ADAM17 was regulated by the tetraspanin CD9 [29] and tissue inhibitor of metallo-proteinase-3 (TIMP3) [30]. Severe hypoxia and Endoplasmic Reticulum Stress (ER) stress could induce expression of ADAM17 [31]. These factors may contribute to the lack of associations between the *NECTIN4* SNPs and the plasma level of released extracellular domain.

We further compared the plasma Nectin-4 levels among the age- and gender- matched normal controls, methadone-free abstinent former heroin users, and patients currently receiving MMT. The average plasma Nectin-4 levels were significantly higher in the MMT patients than the normal controls and the methadone-free abstinent former heroin users. The plasma Nectin-4 levels between abstinent former heroin users and the normal controls who never abused heroin are similar. 15 MMT patients with skin irritation showed a marginally significantly higher plasma level of Nectin-4 than MMT patients without skin irritation. The results suggested that the plasma Nectin-4 is a reversible biomarker for heroin dependent patients and highly related to the status of opioid use in methadone treatment. Details of the regulatory mechanism between the methadone dose and plasma Nectin-4 level warrant more experiments. Nectin-4 protein [17] and μ-opioid receptors [32] have been shown co-localized in the skin keratinocytes. When opioid drugs (either methadone or heroin) were administered in human, the opioids bind to the mu-opioid receptor on keratinocytes, which may result in an increase in the synthesis of metalloproteinase ADAM17/TACE. It is then released into the extracellular space, and lead to break the cell-cell connection through Nectin-4 and increase the plasma Nectin-4 level. *NECTIN3*, another member of the family located on chromosome 3q13.13 with 14 exons (129,686 bp in length), was reported to be associated with neuroticism personality trait in a genome-wide association study with 106,000 subjects [33]. In this personality trait neuroticism study, they also reported that rs4653663, located on chromosome 1q23 near the *ENAH* and *SRP9* genes, was strongly associated with neuroticism personality. It spans 64,875,693 bp near the *NECTIN4* on chromosome 1q23.3 genetic loci (S2 Fig). When the number of subjects was increased to 449,484 in the neuroticism genome-wide association analyses, SNP rs41266050 of *RABGAP1L* was identified as a neuroticism genetic biomarker [34] with an even closer distance 13,465,474 bp toward *NECTIN4*. This suggested that the *NECTIN4* might be one of the candidate genes associated with neuroticism personality trait. This provides indirect evidence for involvement of cell adhesion genes with personality traits.

The limitation of this study includes that the number of MMT patients with skin irritation was small (15 out of 344). However, skin irritation is usually rare in patients under MMT [35]. Our study showed the first report demonstrated that an adhesion molecule *NECTIN4* genetic loci associated with methadone dose, plasma methadone concentration and plasma TNF-α level.

## Conclusion

In summary, this study has identified that the *NECTIN4* gene involving adherens junction interactions pathway is associated with methadone dose using pathway-based genome wide association analyses. Three highly-linked SNPs rs11265549 (intron 1), rs3820097 (intron 2), and rs4656978 (intron 6) located at *NECTIN4* genetic loci were associated with methadone dose, plasma methadone concentration, and plasma TNF-α level. The minor genotype carriers had a lower methadone dose, lower plasma methadone concentrations, higher plasma TNF-α levels than the major genotype carriers. Plasma Nectin-4 level is higher in MMT patients than the normal controls and methadone-free abstinent former heroin users. MMT patients with skin irritation symptoms had a marginally higher plasma Nectin-4 level than MMT patients without skin irritation. The results suggest that plasma Nectin-4 could be an indicator for continued use of opioids including methadone.

## Supporting information

S1 Fig The Haploview analyses of linkage disequilibrium (r^2^) for five SNPs within the *NECTIN4* genetic loci. The number in each square represents r^2^ ⊆100 between two SNPs. The black square without number indicated the r^2^ is equal to 1.
(DOC)

S2 Fig The chromosome position of *NECTIN4* and the distance from SNPs, rs41266050 and rs4653663, which have previously been reported associations with neuroticism personality trait in two separate studies. (DOC)

S1 Table The association analyses between adherens junction interaction pathways and methadone dose (mg/day). (DOC)

S2 Table SNPs in the *NECTIN4* gene loci in the MMT study. (DOC)

S3 Table *NECTIN4* genetic allele types is associated with methadone dosage and plasma *R*,*S*-methadone concentration. (DOC)

S4 Table *NECTIN4* genetic variants are associated with plasma concentrations (ng/mL) of *R*- and *S*-methadone. (DOC)

S5 Table *NECTIN4* genetic allele types are associated with plasma TNF-α level and skin irritation TESS scores. (DOC)

S6 Table General demography in the age- and gender-matched control, medication-free abstinent former heroin users (abstinent), MMT patients with skin irritation, and MMT patients without skin irritation. (DOC)
