# Supplementary material for: Genetic variants in NECTIN4 encoding an adhesion molecule are associated with continued opioid use
Source: PLoS One. 2020 Jun 18;15(6):e0234549. doi: 10.1371/journal.pone.0234549 (PMC7302666; doi:10.1371/journal.pone.0234549)
Supplement: S1 Fig — The number in each square represents r2 ⊆100 between two SNPs. The black square without number indicated the r2 is equal to 1. (DOC) [file pone.0234549.s001.doc]

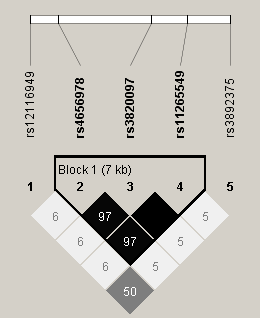


**S1** **Fig.** The Haploview analyses of linkage disequilibrium (r^2^) for five SNPs within the *NECTIN4* genetic loci. The number in each square represents r^2^ 🞨100 between two SNPs. The black square without number indicated the r^2^ is equal to 1.
