# Supplementary material for: Genetic variants in NECTIN4 encoding an adhesion molecule are associated with continued opioid use
Source: PLoS One. 2020 Jun 18;15(6):e0234549. doi: 10.1371/journal.pone.0234549 (PMC7302666; doi:10.1371/journal.pone.0234549)
Supplement: S2 Fig — (DOC) [file pone.0234549.s002.doc]

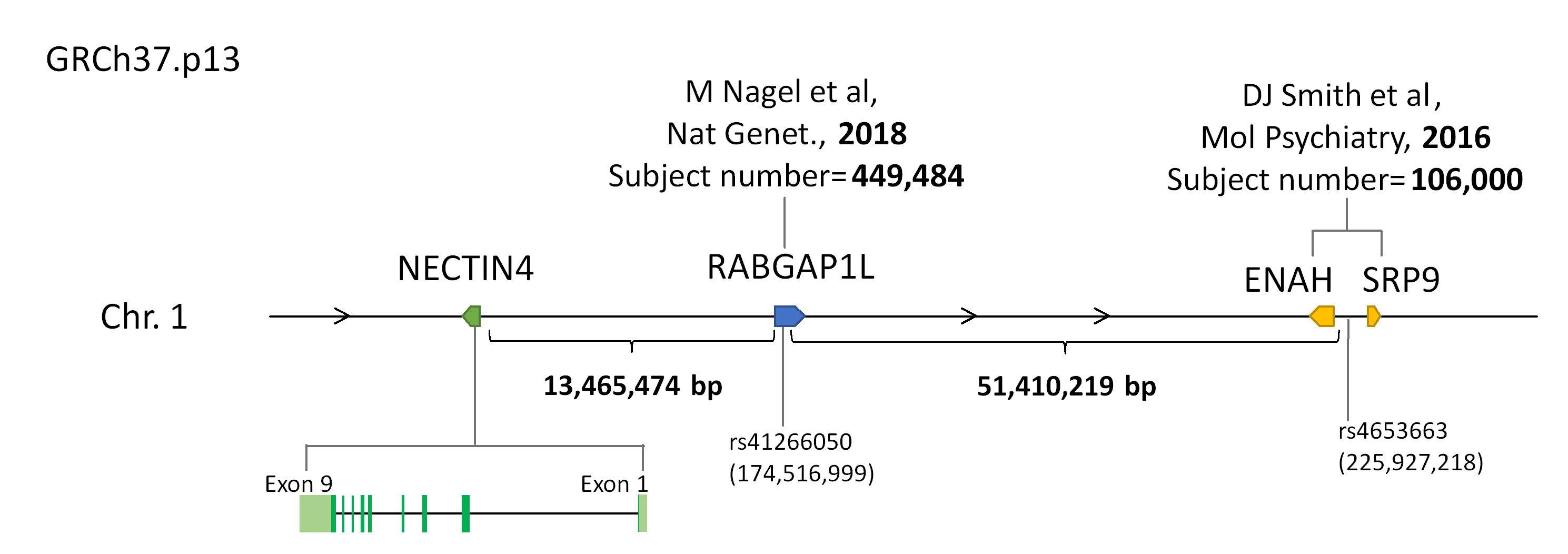


**S2 Fig.** The chromosome position of *NECTIN4* and the distance from SNPs, rs41266050 and rs4653663, which have previously been reported associations with neuroticism personality trait in two separate studies.
