# Supplementary material for: Genetic variants in NECTIN4 encoding an adhesion molecule are associated with continued opioid use
Source: PLoS One. 2020 Jun 18;15(6):e0234549. doi: 10.1371/journal.pone.0234549 (PMC7302666; doi:10.1371/journal.pone.0234549)
Supplement: S1 Table — (DOC) [file pone.0234549.s003.doc]

**S1 Table** The association analyses between adherens junction interaction pathways and methadone dose (mg/day).

| Pathway / Gene | GeneSet  *P*-value | Chromosome | Gene-based *P*-value |
| --- | --- | --- | --- |
| **Adherens junction interactions** | **0.0001** |  |  |
| *NECTIN4* |  | 1 | **0.0008** |
| *CDH2* |  | 18 | **0.007** |
| *CDH13* |  | 16 | **0.016** |
| *CTNND1* |  | 11 | 0.19 |
| *CDH1* |  | 16 | 0.13 |
| *CDH3* |  | 16 | 0.11 |
| *CDH4* |  | 20 | 0.53 |
| *CDH5* |  | 16 | 0.51 |
| *CDH6* |  | 5 | 1.00 |
| *CTNNB1* |  | 3 | 0.70 |
| *CDH8* |  | 16 | 0.89 |
| *CDH7* |  | 18 | 0.67 |
| *CDH9* |  | 5 | 0.29 |
| *NECTIN1* |  | 11 | 0.26 |
| *NECTIN3* |  | 3 | 0.70 |
| *NECTIN2* |  | 19 | 0.32 |
| *CDH24* |  | 14 | 0.89 |
| *CTNNA1* |  | 5 | 0.77 |
| *CDH12* |  | 5 | 0.98 |
| *JUP* |  | 17 | 0.09 |
| *CDH18* |  | 5 | 0.96 |
| *CDH10* |  | 5 | 0.87 |
| *CDH11* |  | 16 | 0.65 |

The pathway-based *P*-values was calculated by the Hybrid set-based test (HYST);

The gene-based *P*-values were calculated by the extended Simes test (GATES);

The pathway-based association threshold was 0.00004.

Bold values indicate P < 0.05.
