# Supplementary material for: Genetic variants in NECTIN4 encoding an adhesion molecule are associated with continued opioid use
Source: PLoS One. 2020 Jun 18;15(6):e0234549. doi: 10.1371/journal.pone.0234549 (PMC7302666; doi:10.1371/journal.pone.0234549)
Supplement: S2 Table — (DOC) [file pone.0234549.s004.doc]

**S2 Table** SNPs in the *NECTIN4* gene loci in the MMT study.

| SNP_ID | Position | Location | HWP | MAF | Alleles |
| --- | --- | --- | --- | --- | --- |
| rs3892375 | 161054054 | Intron 1 | 0.4223 | 0.09 | A:G |
| rs11265549 | 161051526 | Intron 1 | 0.6589 | 0.357 | G:A |
| rs3820097 | 161049358 | Intron 2 | 0.5748 | 0.36 | A:G |
| rs4656978 | 161043764 | Intron 6 | 0.651 | 0.365 | A:G |
| rs12116949 | 161042063 | Exon9, 3' UTR | 0.0823 | 0.132 | C:A |

Localization: according to the isoform of *NECTIN4* mRNA (NM_030916.2).

MAF: Minor allele frequency.

HWP: *P*-value of Hardy-Weinberg equilibrium test.
