# Supplementary material for: Genetic variants in NECTIN4 encoding an adhesion molecule are associated with continued opioid use
Source: PLoS One. 2020 Jun 18;15(6):e0234549. doi: 10.1371/journal.pone.0234549 (PMC7302666; doi:10.1371/journal.pone.0234549)
Supplement: S3 Table — (DOC) [file pone.0234549.s005.doc]

**S3 Table** *NECTIN4* genetic allele types is associated with methadone dosage and plasma *R*,*S*-methadone concentration.

| SNP_ID | Allele type | Methadone dosage (mg/day) | | | | | |  | Plasma *R*,*S*-methadone concentration (ng/mL) | | | | | |
| --- | --- | --- | --- | --- | --- | --- | --- | --- | --- | --- | --- | --- | --- | --- |
|  |  | N | Mean | ± | SD | *P*-value  (Adjusted) | FDR |  | N | Mean | ± | SD | *P*-value | FDR |
| rs3892375  (Intron 1) | A | 626 | 54.68 | ± | 28.44 | 0.099 | 0.099 |  | 626 | 334.11 | ± | 210.67 | 0.248 | 0.248 |
|  | G | 62 | 60.73 | ± | 28.17 | (0.078) |  |  | 62 | 365.78 | ± | 230.66 |  |  |
|  |  |  |  |  |  |  |  |  |  |  |  |  |  |  |
| rs11265549  (Intron 1) | G | 441 | 58.27 | ± | 29.31 | **0.0002** | **0.0006** |  | 441 | 358.98 | ± | 224.25 | **0.0003** | **0.0005** |
|  | A | 245 | 49.90 | ± | 26.10 | (**0.0004**) |  |  | 245 | 297.92 | ± | 184.62 |  |  |
|  |  |  |  |  |  |  |  |  |  |  |  |  |  |  |
| rs12116949  (3' UTR) | C | 597 | 54.29 | ± | 28.53 | **0.027** | **0.034** |  | 597 | 329.77 | ± | 207.72 | **0.023** | **0.029** |
|  | A | 91 | 61.37 | ± | 27.30 | (**0.027**) |  |  | 91 | 384.12 | ± | 237.84 |  |  |
|  |  |  |  |  |  |  |  |  |  |  |  |  |  |  |

SD, standard deviation.

*P*-value, Trend/Correlation analysis of *p*-value.

Parenthesis adjust, *p*-value adjusted body weight with general linear model.

FDR, False Discovery Rate.

Bold values indicate P < 0.05.

rs11265549 was selected as the tagger SNP representing rs3820097 and rs4656978 by the Tagger algorithm in HAPLOVIEW.
