# Supplementary material for: Genetic variants in NECTIN4 encoding an adhesion molecule are associated with continued opioid use
Source: PLoS One. 2020 Jun 18;15(6):e0234549. doi: 10.1371/journal.pone.0234549 (PMC7302666; doi:10.1371/journal.pone.0234549)
Supplement: S4 Table — (DOC) [file pone.0234549.s006.doc]

**S4 Table** *NECTIN4* genetic variants are associated with plasma concentrations (ng/mL) of *R*- and *S*-methadone.

| SNP_ID | Genotype | *R*-methadone (ng/mL) | | | | | |  | *S*-methadone (ng/mL) | | | | | |
| --- | --- | --- | --- | --- | --- | --- | --- | --- | --- | --- | --- | --- | --- | --- |
|  |  | N | Mean | ± | SD | *P*-value | FDR |  | N | Mean | ± | SD | *P*-value | FDR |
| rs3892375  (Intron 1) | AA | 283 | 189.55 | ± | 117.43 | 0.099 | 0.099 |  | 283 | 141.50 | ± | 98.74 | 0.673 | 0.673 |
|  | AG | 60 | 215.89 | ± | 148.66 |  |  |  | 60 | 147.03 | ± | 105.28 |  |  |
|  | GG | 1 | 291.55 | ± | . |  |  |  | 1 | 160.05 | ± | . |  |  |
| rs11265549  (Intron 1) | GG | 144 | 220.56 | ± | 141.38 | **0.0004** | **0.0006** |  | 144 | 160.86 | ± | 109.90 | **0.002** | **0.003** |
|  | AG | 153 | 182.09 | ± | 108.00 |  |  |  | 153 | 134.64 | ± | 89.02 |  |  |
|  | AA | 46 | 154.44 | ± | 95.31 |  |  |  | 46 | 112.20 | ± | 90.86 |  |  |
| rs12116949  (3' UTR) | CC | 255 | 181.64 | ± | 109.37 | **0.003** | **0.003** |  | 255 | 137.42 | ± | 96.76 | 0.172 | 0.215 |
|  | AC | 87 | 233.34 | ± | 153.15 |  |  |  | 87 | 159.25 | ± | 106.91 |  |  |
|  | AA | 2 | 134.78 | ± | 27.12 |  |  |  | 2 | 65.15 | ± | 32.24 |  |  |

SD, standard deviation.

*P*-value, Trend/Correlation analysis of *p*-value.

FDR, False Discovery Rate.

Bold values indicate *P* < 0.05.

rs11265549 was selected as the tagger SNP representing rs3820097 and rs4656978 by the Tagger algorithm in HAPLOVIEW.
