# Supplementary material for: Genetic variants in NECTIN4 encoding an adhesion molecule are associated with continued opioid use
Source: PLoS One. 2020 Jun 18;15(6):e0234549. doi: 10.1371/journal.pone.0234549 (PMC7302666; doi:10.1371/journal.pone.0234549)
Supplement: S5 Table — (DOC) [file pone.0234549.s007.doc]

**S5 Table** *NECTIN4* genetic allele types are associated with plasma TNF-α level and skin irritation TESS scores

| SNP_ID | Allele type | Plasma TNF-α (pg/ml) | | | | | |  | Skin irritation | | | | | |
| --- | --- | --- | --- | --- | --- | --- | --- | --- | --- | --- | --- | --- | --- | --- |
|  |  | N | Mean | ± | SD | *P*-value | FDR |  | N | Mean | ± | SD | *P*-value | FDR |
| rs3892375  (Intron 1) | A | 616 | 10.64 | ± | 9.01 | 0.374 | 0.374 |  | 30 | 1.47 | ± | 0.82 | - | - |
|  | G | 62 | 9.61 | ± | 4.00 |  |  |  | 0 | . | ± | . |  |  |
|  |  |  |  |  |  |  |  |  |  |  |  |  |  |  |
| rs11265549  (Intron 1) | G | 437 | 9.89 | ± | 5.96 | **0.009** | **0.021** |  | 18 | 1.17 | ± | 0.51 | **0.014** | **0.019** |
|  | A | 239 | 11.71 | ± | 12.11 |  |  |  | 12 | 1.92 | ± | 1.00 |  |  |
|  |  |  |  |  |  |  |  |  |  |  |  |  |  |  |
| rs12116949  (3' UTR) | C | 587 | 10.67 | ± | 9.12 | 0.339 | 0.374 |  | 27 | 1.52 | ± | 0.85 | 0.298 | 0.298 |
|  | A | 91 | 9.74 | ± | 4.90 |  |  |  | 3 | 1.00 | ± | 0.00 |  |  |
|  |  |  |  |  |  |  |  |  |  |  |  |  |  |  |

SD, standard deviation.

*P*-value, Trend/Correlation analysis of *p*-value.

FDR, False Discovery Rate.

Bold values indicate P < 0.05.

rs11265549 was selected as the tagger SNP representing rs3820097 and rs4656978 by the Tagger algorithm in HAPLOVIEW.
