# Supplementary material for: Genetic variants in NECTIN4 encoding an adhesion molecule are associated with continued opioid use
Source: PLoS One. 2020 Jun 18;15(6):e0234549. doi: 10.1371/journal.pone.0234549 (PMC7302666; doi:10.1371/journal.pone.0234549)
Supplement: S6 Table — (DOC) [file pone.0234549.s008.doc]

**S6 Table** General demography in the age- and gender-matched control, medication-free abstinent former heroin users (abstinent), MMT patients with skin irritation, and MMT patients without skin irritation.

|  | Control **^1^** | | | |  | Medication-free abstinent **^2^** | | | |  | MMT patients | | | | | | | | |  |  |
| --- | --- | --- | --- | --- | --- | --- | --- | --- | --- | --- | --- | --- | --- | --- | --- | --- | --- | --- | --- | --- | --- |
|  |  |  |  |  |  |  |  |  |  |  | Skin irritation **^3^** | | | |  | No skin irritation **^4^** | | | |  |  |
| Variable | N | Mean | ± | SD |  | N | Mean | ± | SD |  | N | Mean | ± | SD |  | N | Mean | ± | SD | *P*-value | Post hoc |
| Age | 51 | 34.14 | ± | 6.05 |  | 83 | 35.87 | ± | 6.00 |  | 15 | 38.27 | ± | 7.35 |  | 174 | 35.36 | ± | 5.60 | 0.12 |  |
| BMI | 51 | 24.60 | ± | 3.89 |  | 82 | 26.28 | ± | 3.44 |  | 15 | 21.99 | ± | 2.82 |  | 173 | 23.78 | ± | 3.16 | **<0.0001** | 2>1, 3, 4**;  1, 4>3* |
| Gender |  |  |  |  |  |  |  |  |  |  |  |  |  |  |  |  |  |  |  | 0.09 |  |
| Male | 43 | ( 84.31 % ) | | |  | 80 | ( 96.39 % ) | | |  | 14 | ( 93.33 % ) | | |  | 153 | ( 87.93 % ) | | |  |  |
| Female | 8 | ( 15.69 % ) | | |  | 3 | ( 3.61 % ) | | |  | 1 | ( 6.67 % ) | | |  | 21 | ( 12.07 % ) | | |  |  |
| Nectin-4 (pg/ml) | 51 | 164.91 | ± | 54.54 |  | 83 | 153.23 | ± | 41.71 |  | 14 | 259.67 | ± | 67.48 |  | 174 | 224.03 | ± | 58.36 | **<0.0001** | 3, 4>1, 2** |
| Cotinine (ng/ml) | 51 | 90.40 | ± | 113.74 |  | 83 | 4.25 | ± | 26.08 |  | 15 | 423.62 | ± | 169.01 |  | 174 | 394.34 | ± | 184.39 | **<0.0001** | 1, 3, 4>2**;  3, 4>1** |
| Liver function test |  |  |  |  |  |  |  |  |  |  |  |  |  |  |  |  |  |  |  |  |  |
| AST (U/L) | 50 | 24.94 | ± | 8.69 |  | 83 | 47.82 | ± | 35.99 |  | 14 | 58.07 | ± | 50.07 |  | 168 | 50.57 | ± | 43.21 | **<0.0001** | 2, 3, 4>1** |
| ALT (U/L) | 50 | 27.90 | ± | 18.28 |  | 83 | 77.95 | ± | 80.93 |  | 14 | 86.43 | ± | 95.07 |  | 171 | 59.14 | ± | 65.42 | **<0.0001** | 2, 3, 4>1** |
| γ-GT (U/L) | 50 | 39.76 | ± | 46.97 |  | 83 | 40.14 | ± | 35.49 |  | 13 | 50.69 | ± | 45.37 |  | 159 | 63.56 | ± | 95.13 | 0.10 |  |
| HIV |  |  |  |  |  |  |  |  |  |  |  |  |  |  |  |  |  |  |  | **<0.0001** | 3, 4>1**; 4>2 *; 2>1* |
| Negative | 50 | ( 100.00 % ) | | |  | 75 | ( 90.36 % ) | | |  | 11 | ( 78.57 % ) | | |  | 128 | ( 74.42 % ) | | |  |  |
| Positive | 0 | ( 0.00 % ) | | |  | 8 | ( 9.64 % ) | | |  | 3 | ( 21.43 % ) | | |  | 44 | ( 25.58 % ) | | |  |  |
| HCV |  |  |  |  |  |  |  |  |  |  |  |  |  |  |  |  |  |  |  | **<0.0001** | 2, 3, 4>1**;  4>2 * |
| Negative | 49 | ( 100.00 % ) | | |  | 16 | ( 19.51 % ) | | |  | 0 | ( 0.00 % ) | | |  | 14 | ( 8.33 % ) | | |  |  |
| Positive | 0 | ( 0.00 % ) | | |  | 66 | ( 80.49 % ) | | |  | 13 | ( 100.00 % ) | | |  | 154 | ( 91.67 % ) | | |  |  |
| Continuous variable test by Kruskal Wallis test for four groups and Mann-Whitney U test for multiple comparison (post hoc); the categorical variable analysis by Chi-square test. | | | | | | | | | | | | | | | | | | | | | |
| AST, Aspartate aminotransferase. ALT, Alanine aminotransferase. γ-GT, Gamma-glutamyl transpeptidase. HIV, Human Immunodeficiency Virus. HCV, Hepatitis C virus antibody. | | | | | | | | | | | | | | | | | | | | | |
| *: *P*<0.05; **: *P*<0.01 | | | | | | | | | | | | | | | | | | | |  |  |
